# Minimum acceptable diet and its predictors among children aged 6‐23 months in Ethiopia. A multilevel cloglog regression analysis

**DOI:** 10.1111/mcn.13647

**Published:** 2024-03-26

**Authors:** Tegene Atamenta kitaw, Befkad Derese Tilahun, Biruk Beletew Abate, Ribka Nigatu Haile

**Affiliations:** ^1^ Department of Nursing, College of Health Science Woldia University Woldia Ethiopia

**Keywords:** Children, Ethiopia, Minimum acceptable diet predictors

## Abstract

Despite significant progress made previously and the recognized health benefits of optimal feeding practices, ensuring a minimum acceptable diet in developing countries like Ethiopia remains a formidable challenge. Additionally, there is a scarcity of data in this area. Therefore, our study aims to identify predictors of a minimum acceptable diet using a powerful tool called complementary log‐log regression analysis. Thus, it contributes to accelerating the pathway of ending child undernutrition thereby promoting optimal child health. A multilevel analysis was conducted among a weighted sample of 1427 children aged 6‐23 months using the 2019 Ethiopian Demographic Health Survey (EDHS). The EDHS sample was stratified and selected in two stages. A minimum acceptable diet is defined as a composite of children fed with both minimum dietary diversity and minimum meal frequency. Data extraction took place between August 1 and 30, 2023. We used STATA software version 17 for data analysis. A complementary log‐log regression model was fitted to identify significant predictors of the minimum acceptable diet. A p‐value of ≤0.05 was used to declare statistically significant predictors. Only 10.44% (95CI: 8.90‐12.15) of the children meet the minimum acceptable diet. Child aged (18‐23 month) (AOR = 1.78, 95CI:1.14‐2.78)], mother's educational level (secondary and above education) (AOR = 279,95CI: 1.51‐5.15), number of children three and above [(AOR = 0.78,95CI: 0.53‐0.94], institutional delivery [AOR = 1.77,95CI: 1.11‐3.11], having postnatal‐check‐up [AOR = 2.33,95CI: 1.59‐3.41] and high community poverty level (AOR = 0.49,95CI: 0.29‐0.85) were found to be predictors of minimum acceptable diet. In Ethiopia, only one in ten children achieve a minimum acceptable diet. Which is lower than the global report findings (16%). Enhancing maternal education programs and promoting family planning strategies to reduce household size are essential. Besides, encouraging institutional deliveries and postnatal check‐ups are also recommended. It is necessary to implement targeted interventions for poverty reduction in communities to ensure that families can afford nutritious diets for their children.

## Key messages

- This study aimed to identify predictors of a minimum acceptable diet among children aged 6‐23 months.
- Analysis revealed that only 10.44% of children met the minimum acceptable diet criteria.
- Factors such as child age, mother's educational level, number of children in the household, place of delivery, postnatal checkup 2 months after delivery, and community poverty level emerged as significant predictors of achieving a minimum acceptable diet.
- These findings underscore the multifactorial nature of dietary adequacy in early childhood and highlight the importance of targeted interventions to improve nutritional outcomes for young children.

## Graphical abstract

Minimum acceptable diet coverage among children aged 6‐23 months in Ethiopia, EDHS 2019

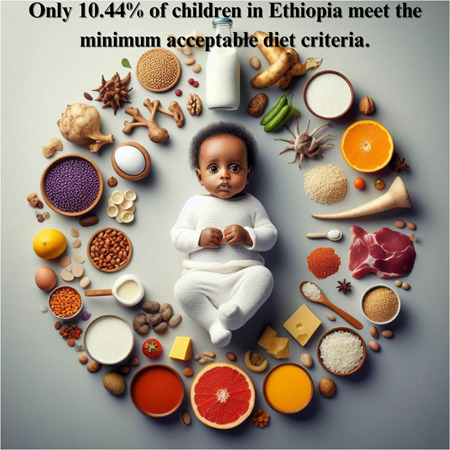

## 1 INTRODUCTION

Sufficient nutrition is predominant for optimal physical and cognitive development of the child. Children's morbidity and mortality associated with undernutrition are high (Kirkpatrick et al., 2010). The minimum acceptable diet (MAD) is a crucial indicator for evaluating infant and young child feeding (IYCF) practices among children aged 6 to 23 months. It encompasses two essential components: minimum dietary diversity (MDD) and minimum meal frequency (MMF) (World Health Organization, 2010). MDD ensures that a child's diet includes a variety of food groups from different nutritional categories, while MMF ensures that the child receives an adequate number of meals and snacks throughout the day (World Health Organization, 2011).

Maintaining a MAD during infancy and early childhood is crucial for ensuring optimal growth and development (Das et al., 2016). Failure to achieve MAD places young children at heightened risk of undernutrition, particularly stunting and micronutrient deficiencies. These nutritional inadequacies can lead to increased morbidity and mortality (WHO U, USAID A, AED U, 2008), underscoring the critical importance of providing adequate and balanced diets during this critical period of life.

Undernutrition, a state of inadequate nutrient intake, is a significant contributor to child mortality, claiming the lives of nearly half of all children under the age of five (Unicef, 2018). A substantial proportion of childhood fatalities, amounting to almost one‐third, occur in the African Region and are directly attributable to malnutrition (World Health Organization, 2020).

In Ethiopia, a significant proportion of child mortality, amounting to 28%, is linked to inadequate nutrition (UNICEF Global, 2023). Only 4% of children between the ages of 6 and 23 months receive a diverse diet encompassing four or more food groups. Moreover, the frequency of feeding sessions is also suboptimal, with only 45% of children in this age group being fed at least thrice daily (UNICEF Ethiopia, 2018). This alarming situation is reflected in the low prevalence of optimal feeding practices among young children.

Previous research identified various factors influencing the likelihood of children achieving a minimum acceptable diet (MAD). These factors include residential location (Kambale et al., 2021), maternal age (Acharya et al., 2021), maternal education level (Birie et al., 2021), socioeconomic status (Belay et al., 2022), exposure to media messaging about healthy eating (Gizaw & Tesfaye, 2019), maternal employment status (Molla et al., 2021), place of delivery (Feleke & Mulaw, 2020), frequency of antenatal care attendance, and participation in postnatal care services (Shaun et al., 2023).

The Sustainable Development Goals (SDGs) have targeted reducing newborn and under‐five mortality by 2030, aiming for as low as 12 and 25 deaths per 1 000 live births, respectively (Colglazier, 2015). Achieving these targets hinges significantly on ensuring that children consume a minimum acceptable diet (MAD). In line with this global initiative, Ethiopia's Ministry of Health has also adopted a comprehensive plan to address malnutrition and diet‐related public health issues, recognizing their crucial role in reducing under‐five mortality. The Ministry's strategy promotes MAD among children as a critical intervention for achieving this goal (Ministry of Health of Ethiopia, 2022).

Despite the enormous progress made previously and the known health benefits of optimal feeding practices, it has become a significant challenge to increase the number of children getting a minimum acceptable diet in developing countries like Ethiopia. Thus, recent data regarding minimum acceptable is crucial for public health practitioners and policymakers in supporting efforts towards strengthening child nutrition programs and ending all types of malnutrition in the children. Furthermore, the study was carried out using recent data (2019) from the Ethiopia Demographic and Health Survey (EDHS), which is crucial in providing up‐to‐date information on national improvements regarding child feeding practices. Understanding factors determining MAD is also essential in designing strategies for improving child nutrition.

Most studies regarding minimum acceptable diet in Ethiopia are limited to specific districts/areas. Besides, most of the studies use logistic regression to identify factors affecting MAD. According to previous reports, only 7% of the children achieve MAD (21). When the occurrence of a particular event is very small or substantial, complementary log‐log regression is the most powerful and versatile statistical modelling technique to identify predictors of the outcomes (Akif Mustafa, 2019). Consequently, this study employed multilevel mixed‐effects complementary log‐log regression analysis to determine predictors of MAD.

## 2 METHODS

### 2.1 Study setting, study period and data source

According to forecasts from trading economics and data from recent census figures, the total population of Ethiopia was 115.0 million by 2020 (“Ethiopia Population ‐ 2021 Data ‐ 2022 Forecast ‐ 1960‐2020 Historical ‐ Chart ‐ News”, 2022). The EDHS 2019 final report includes data at the country level from the nine regional states and two municipal administrations. The administrative levels were divided into zones, woreda (districts), and so forth. A multilevel analysis was conducted among children between 6 and 23 months. The EDHS collects pertinent information mainly regarding maternity health care utilization, marriage and sexual behaviour, child feeding practice, children and women's dietary condition, and children's and adult mortality. The study was done by using the 2019 EDHS data set. Data collection for EDHS 2019 was carried out from March to June 2019 (Ethiopian Public Health Institute EPHI and ICF, 2019).

### 2.2 Data extraction, population

First, the project proposal was sent to the Demographic and Health Surveys (DHS) program with a request to use the data of the EDHS. After a detailed review process, the DHS program accepted the proposal and granted access with an approval letter to use the survey datasets. Data extraction was done to select children aged 6‐23 months. The data extraction was conducted between August 1 and 30, 2023. All children aged 6‐23 months were the source population, whereas all children aged 6‐23 months in the selected enumeration area were the study population.

### 2.3 Sampling methods

The EDHS 2019 sample was stratified and selected in two levels. A total of 21 sampling strata were produced after stratifying each region into urban and rural areas. Using probability proportion, 305 enumeration areas (93 from urban and 212 from rural) were selected in the first stage. A newly formed household listing was used in the second stage to choose a set number of 30 households per cluster with an equal probability of systematic selection. Sample allocation was done to verify that survey precision was equivalent across regions. Of the enumeration areas 35 were selected from the three largest regions, 25 enumeration areas were selected from eight other regions (including two city administrations). The complete sampling procedure is available in the EDHS 2019 final report (Ethiopian Public Health Institute ‐ EPHI, Federal Ministry of Health ‐ FMoH, ICF, 2021). In the current study, a total of 1427 sample of children aged 6‐23 month participated.

### 2.4 Study variables

The dependent variable is minimum acceptable diet. This study considered different independent variables to identify determinants of minimum acceptable diet. (Table 1). The community‐level variables are constructed using individual‐level factors at the cluster (community) level, thereby classified as lower or higher by using medial value as a cut‐off point if the distribution is not normal. Furthermore, the distribution was checked by using a histogram.

**Table 1:** List of independent variables for the assessment of predictors of minimum acceptable diet in Ethiopia.

Variable	Descriptions (classification)
Sex of the child	Male or female
Age of the child	6‐11 month, 12‐17 month, 18‐23 month
Mother age	15–24,25–34, 35–49
Residence status	Urban or Rural
Region	Larger central: Tigray, Amhara, Oromia, SNNPR Small peripherals: Benishangul, Gambela, Afar, Somali Metropolis: Harari, Addis Ababa, Dire Dawa (Mare et al., 2022; Teshale & Tesema, 2020).
Mother Educational status	No education, Primary, Secondary or higher
Wealth index	Poor, Middle, and Rich
Marital status	Not married or married
Antenatal	No ANC visit, 1–3 or 4 and above
Place of delivery	Home or health institution
Type of birth	Single or twin and above
Family size	2‐5, 6‐10, >10
Sex of head of household	Male or Female
Birth order	First, 2nd or 3rd, 4th and above
Number of children	One or two, above two
Community poverty level	Low or High
Community women education level	Low or High
Media access (television)	Yes or No
Media access (radio)	Yes or No

### 2.5 Definitions

Minimum acceptable diet is defined as a composite of children fed with a minimum dietary diversity and a minimum meal frequency (EPHI I, 2019). Minimum dietary diversity: children aged 6‐23 months who received a minimum of five out of eight food groups during the previous day (World Health Organization, 2021). Minimum meal frequency: For breastfed children receiving solid, semisolid, or soft food at least twice a day for infants aged 6‐8 months and at least three times daily for children aged 9‐23 months. For non‐breastfed children, aged 6‐23 months aged receiving solid, semisolid, or soft food or milk feeds at least four times a day (World Health Organization, 2021).

### 2.6 Data processing and analysis

Data was extracted from the EDHS 2019 individual record folder using STATA version 17. Sorting and listing were employed to identify any missing values. Frequency and percentage were computed for descriptive statistics. Date weighting, cleaning, editing, and recording were carried out.

### 2.7 Multilevel mixed effect complementary log‐log regression analysis

#### 2.7.1 Complementary log‐log regression analysis

Complementary log‐log models represent an alternative to logistic regression and probit analysis for binary response variables. Complementary log‐log models are frequently used when the probability of an event is very small or very large. Unlike logit and probit, the complementary log‐log function is asymmetrical. The assumption of logistic regression might not hold where the probability of success or event occurrence is either low or high. In this scenario, complementary log‐log regression will be the most powerful tool to identify predictors of the outcome variable. The model can be written as (Akif Mustafa, 2019):

$$P(Y=1)=\frac{1}{1-\exp\left[-\exp\left(\sum_{i=1}^{k}\beta_{i}X_{i}\right)\right]}$$

Multilevel mixed‐effect complementary log‐log regression analysis was computed to identify significant. Multilevel modelling is a statistical model used to analyze data drawn from different levels. Its equation can be indicated as

$$\mathrm{Logit}\left(\pi_{ij}\right)=\beta_{0}+\beta x_{ij}+U_{j};$$

β_0_ indicates intercepts, β is individual level factors (unknown) whereas U_j_ is Gaussian random effects (mutually independent) (Tamirat et al., 2023).

A multilevel model was fitted because of the hierarchical nature of the EDHS data. Four models were considered. The first (null) model considers only the dependent variable to explore the degree of cluster variation on the minimum acceptable diet. The second model and the third contain individual‐level factors and community‐level factors. The fourth model is adjusted for both individual and community levels concurrently. Adjusted odds ratio (AOR) and respective 95% confidence interval were computed to identify significant predictors of a minimum acceptable diet. A variance inflation factor and tolerance value were used to check the exitances of multicollinearity between variables. A VIF above 4 or tolerance below 0.25 indicated that multicollinearity might exist (Kitaw & Haile, 2022, 2023). To estimate the variation between clusters, Proportional change variance (PCV), intra‐class correlation (ICC), and Median odd ratio (MOR) were computed.

ICC shows the degree of heterogeneity of achieving a minimum acceptable diet between clusters and was calculated as:

$$\mathrm{ICC}=\frac{\sigma^{2}}{\left(\sigma^{2}+\sigma_{b}^{2}\right)},$$

where σ^2^ represent community level variance, $\sigma_{b}^{2}$ indicates individual level variance (Rabe‐Hesketh & Skrondal, 2012). $\sigma_{b}^{2}=\frac{\pi^{2}}{3}$

MOR is the median variation of odds ratio between high‐risk areas of a minimum acceptable diet and low risk during randomly picking out of clusters. It is calculated as:

$$\mathrm{MOR}=e^{\left[\sqrt{\left(2*V_{A}\right)0.6745}\right]}=e^{\left[0.95*\sqrt{V_{A}}\right]},$$

where VA represents the area level variance (Merlo et al., 2005).

PCV measures the total variation in achieving minimum acceptable diet attributable to factors in successive models. It is computed as:

$$\mathrm{PCV}=\frac{V_{\mathrm{null}}-V_{A}}{V_{\mathrm{null}}}\;*\;100\%,$$

where V_null_ is the variance in the null model and V_A_ is the variance in the successive model (Liyew & Teshale, 2020).

### 2.8 Ethics approval and consent to participate

The EDHS 2019 was ethically reviewed by the National Research Ethics Review Committee (NRERC) of the Ethiopian Ministry of Science and Technology. As described in the survey final report, involvement in the survey program was voluntary, and verbal agreement (informed consent) was also taken (Ethiopian Public Health Institute EPHI and ICF, 2019). The project proposal underwent a submission process and was subsequently assessed by the DHS team. Following this evaluation, we received a formal letter granting consent and providing access to the dataset for utilization in our study. This letter likely outlined the terms and conditions of data usage, including any restrictions, permissions, or requirements for ethical considerations and data security.

## 3 RESULTS

### 3.1 Individual level characteristics of study participants

A total of 1427 weighted sample of children aged 6‐23 months were included to explore the predictors of a minimum acceptable diet. Among mother with no education, 697(48.84%), only 1.89% of their children gets a minimum acceptable diet. Regarding wealth status, from 661(46.32%) children within the poor wealth level, only 1.61% of the children met the minimum acceptable diet. Only 3.85% of children achieve a minimum acceptable diet in households with a number of children of three and above. (Table 2).

**Table 2:** Individual level characteristics of study participants EDHS 2019, (weighted n = 1427).

Variables	Categories	Minimum acceptable diet		Total (weighted), N
		No	Yes	
Sex of the child	Male	653 (45.76%)	74 (5.19%)	727 (50.95%)
	Female	625 (43.80%	75 (5.26%)	700 (49.05%)
Age of the child	6‐11 months	458 (32.10%)	33 (2.31%)	491 (34.41%)
	12‐17 months	469 (32.87 (%)	64 (4.48%)	533 (37.35%)
	18‐23 months	351 (24.60%)	52 (3.64%)	403 (28.24%)
Age of the mother (year)	15‐24	398 (27.89%)	43 (3.01%)	441 (30.90%)
	25‐34	673 (47.16%)	88 (6.17%)	761 (53.33%)
	35‐49	207 (14.51%)	18 (1.26%)	225 (15.77%)
Educational status	No education	670 (46.95%)	27 (1.89%)	697 (48.84%)
	Primary education	434 (30.41%)	63 (4.41%)	497 (34.83%)
	Secondary education	103 (7.22%)	29 (2.03%)	132 (8.25%)
	Higher education	71 (4.98%)	30 (2.10%)	101 (7.08%)
Marital status	Married	1198 (83.95%)	137 (9.60%)	1335 (93.55%)
	Not married	80 (5.61%)	12 (0.84%)	92 (6.45%)
Wealth index level	Poor	638 (44.71%)	23 (1.61%)	661 (46.32%)
	Middle	187 (13.1%)	26 (1.82%)	213 (14.93%)
	Rich	453 (31.74%)	100 (7.01%)	553 (38.75%)
Family size	2‐5	627 (43.94%)	89 (6.24%)	716 (2018%)
	6‐10	606 (42.47)	57 (3.99%)	663 (46.46%)
	>10	45 (3.15%)	3 (0.21%)	48 (3.36%)
Sex of head of household	Male	991 (69.45%)	118 (8.27%)	1109 (77.72%)
	Female	287 (20.11%)	31 (2.17%)	318 (22.28%)
Birth order	First	265 (18.57%)	57 (3.99%)	322 (22.56%)
	2nd or 3rd	478 (33.5%)	53 (3.71%)	531 (37.21%)
	4th or above	535 (37.49%)	39 (2.73%)	574 (40.22%)
Number of children	One or two	564 (39.52%)	94 (56.59%)	658 (46.11%)
	Above two	714 (50.04%)	55 (3.85%)	769 (53.89%)
Media access (television)	Yes	1054 (73.86%)	79 (5.54%)	1133 (79.40%)
	No	224 (15.70%	70 (4.91%)	294 (20.60%)
Media access (radio)	Yes	963 (67.48%)	84 (5.89%)	1047 (73.37%)
	No	315 (22.07%)	65 (4.56%)	380 (26.63%)
Place of delivery	Home	590 (41.35%)	22 (1.54%)	612 (42.89%)
	Health institution	688 (48.21%	127 (8.90%	815 (57.11%)
ANC	No ANC visit	345 (24.18%)	16 (1.12%)	361 (25.30%)
	1‐3 visits	429 (30.06%)	32 (2.24%)	461 (32.31%)
	4 and above visits	504 (35.32%)	101 (7.08%)	605 (42.40%)
PNC check‐up within 2 months	No	1133 (79.40%	109 (7.64%)	1242 (87.04%)
	Yes	145 (10.16%)	40 (2.80%)	185 (12.96%)

### 3.2 Community level characteristics of study participants

The majority of the participants (73.8%) reside in rural areas. Among communities with high poverty levels (56.97%), only 2.45% of the children receive the minimum acceptable diet. Regarding the educational level, 974(68.26%) of the mothers were from a low community education level. (Table 3).

**Table 3:** Community level characteristics of study participants EDHS 2019, (weighted n = 1427).

Variable	Categories	Minimum acceptable diet		Total (weighted), N
		No	Yes	
Place of residence	Urban	298 (20.88%)	76 (5.33%)	374 (26.21%)
	Rural	980 (68.68%)	73 (5.12%)	1053 (73.79%)
Region	Large central	536 (37.56%)	64 (4.48%)	600 (42.05%)
	Small peripheral	491 (34.41%)	29 (2.03%)	520 (36.44%)
	Metropolitan	251 (17.59%)	56 (3.92%)	307 (21.51%)
Community poverty level	Low	500 (35.04%)	114 (7.99%)	614 (43.03%)
	High	778 (54.52%)	35 (2.45%)	813 (56.97%)
Community women education level	Low	916 (64.19%)	58 (4.06%)	974 (68.26%)
	High	362 (25.37%)	91 (6.38%)	453 (31.74%)

### 3.3 Multilevel mixed effect complementary log‐log regression analysis

#### 3.3.1 Multicollinearity test

A variance inflation factor and tolerance value were used to check the exitances of multicollinearity between variables. A VIF above 4 or tolerance below 0.25 indicated that multicollinearity might exist. In this study, the maximum VIF was 3.02, with a mean VIF of 1.87, and the minimum tolerance value was 0.33. Thus, there is no multicollinearity between covariates. (Table 4).

**Table 4:** VIF and tolerance test to check the existence of multicollinearity between covariates.

Variable	VIF	1/VIF
Birth order	3.02	0.331576
Wealth index level	2.98	0.335083
Number of children	2.72	0.368270
Community poverty level	2.39	0.417604
Medica access (Television)	2.25	0.443531
Residence	2.04	0.489503
Mother's educational level	1.92	0.519705
Place of delivery	1.65	0.604231
Community education level	1.64	0.609582
Antenatal care utilization	1.49	0.670114
Family size	1.44	0.696010
Region	1.29	0.777891
Medica access (radio)	1.15	0.869045
Post natal care utilization	1.04	0.963408
Age of the child	1.01	0.986180
Mean VIF	1.87	

### 3.4 Model comparison and random effect

The value of ICC in the null model shows that 20.4% of the difference in receiving a minimum acceptable diet among children is due to cluster (enumeration area) difference. Furthermore, the MOR value in the null model revealed that 2.40 times the odds of difference in the minimum acceptable diet between study subjects is attributed to differences in clusters. The PCV in the final model explained that 18.69% variation in achieving a minimum acceptable diet is because of individual and community‐related factors. DIC and Log‐likelihood were computed for model comparison. A model with a small DIC and high Log‐likelihood was declared the best‐fitted model. Thus, Model III was the best‐fitted model (DIC = 827.22, Log‐likelihood = −394.44686). (Table 5).

**Table 5:** Result of model comparison and random effect to determine predictors of minimum acceptable diet EDHS 2019.

Parameter	Null model	Model I	Model II	Model III
ICC	20.4%	16.0%	19.3%	15.5%
MOR	2.40	2.12	2.32	2.09
PCV	Ref	16.07%	7.02	18.69%
Model comparison				
DIC	957.1498	829.8868	861.5609	827.2226
Log‐likelihood	−455.00784	−400.03463	−418.33229	−394.44686

Abbreviations: DIC, Deviance information criterion; ICC, Intracluster correlatio; MOR, Median odds ratio; PCV, Proportional change in variance.

### 3.5 Predictors of minimum acceptable diet

The result of multilevel mixed effect complementary log‐log regression analysis (Model III) revealed that the age of the child, educational status, wealth index level, number of children, place of delivery, PNC checkup within 2 months and community poverty level were found to be predictors of a minimum acceptable diet.

The odds of achieving a minimum acceptable diet among children aged 12‐17 months and 18‐23 months were 1.85 (AOR = 1.91,95CI: 1.25‐2.93) and 1.78 (AOR = 178, 95CI:1.14‐2.78), respectively. Mothers who attend primary and secondary education were 2.33 (AOR = 2.33,95CI: 141‐3.84) and 2.79 times (AOR = 279,95CI: 1.51‐5.15) more likely to give a minimum acceptable diet for their children, respectively. In households with the number of children three and above were, 22% (AOR = 0.78,95CI: 0.53‐0.94) less likely to achieve the minimum acceptable diet compared to households with less than two children. Mothers who gave birth in health institutions were 1.77 times (AOR = 1.77,95CI: 1.11‐3.11) more likely to give a minimum acceptable diet for their children than the reverse group. The child of a mother with a postnatal checkup within 2 months after delivery had a 2.33 (AOR = 2.33,95CI: 1.59‐3.41) times higher likelihood in meeting the minimum acceptable diet. In a community with a high poverty level, the likelihood of achieving a minimum acceptable diet is reduced by nearly 50% (AOR = 0.49,95CI: 0.29‐0.85). (Table 6).

**Table 6:** Multivariable multilevel mixed effect complementary log‐log regression analysis of predictors of a minimum acceptable diet EDHS 2019, (weighted n = 1427).

Variables	Model I AOR(95% CI)	Model II AOR(95% CI)	Model III AOR (95% CI)
Age of the child			
6‐11 month	*Ref*.		*Ref*.
12‐17 month	1.85 (1.21‐2.83)		1.91 (1.25‐2.93)**
18‐23 month	1.78 (1.15‐2.78)		1.78 (1.14‐2.78)*
Educational status			
No education	*Ref*.		*Ref*.
Primary education	2.34 (1.43‐3.85)		2.33 (1.41‐3.84)***
Secondary and above	3.09 (1.76‐5.42)		2.79 (1.51‐5.15)***
Wealth index level			
Poor	*Ref*.		*Ref*.
Middle	2.43 (1.35‐4.37)		1.70 (0.89‐3.26)
Rich	1.70 (0.93‐3.10)		0.92 (0.45‐1.88)
Family size			
2‐5	*Ref*.		*Ref*.
6‐10	0.87 (0.58‐1.31)		0.92 (0.61‐1.38)
>10	0.63 (0.19‐2.09)		0.65 (0.19‐2.16)
Birth order			
First	*Ref*.		*Ref*.
2nd or 3rd	0.71 (0.47‐1.090)		0.69 (0.45‐1.07)
4th or above	1.18 (0.55‐2.52)		1.19 (0.56‐2.54)
Number of children			
One or two	*Ref*.		*Ref*.
Three or above	0.85 (0.47‐0.97)		0.78 (0.53‐0.94)*
Media access (television)			
No	*Ref*.		*Ref*.
Yes	1.66 (1.07‐2.58)		1.24 (0.74‐2.09)
Media access (radio)			
No	*Ref*.		*Ref*.
Yes	1.30 (0.93‐1.83)		1.32 (0.93‐1.86)
Place of delivery			
Home	*Ref*.		*Ref*.
Health institution	1.87 (1.05‐3.23)		1.77 (1.11‐3.11)*
ANC			
No ANC visit	*Ref*.		*Ref*.
1‐3 visits	1.59 (1.31‐2.14)		0.58 (0.29‐1.12)
4 and above visits	1.979 (1.52‐2.83)		0.91 (0.48‐1.74)
PNC checkup within 2 months			
No	*Ref*.		*Ref*.
Yes	2.28 (1.56‐3.25)		2.33 (1.59‐3.41)***
Place of residence			
Urban		*Ref*.	*Ref*.
Rural		0.65 (0.43‐0.970	0.734 (0.45‐1.19)
Region			
Large central		*Ref*.	*Ref*.
Small peripheral		0.69 (0.44‐1.10)	0.85 (0.53‐1.35)
Metropolitan		0.94 (0.62‐1.41)	0.92 (0.59‐1.43)
Community poverty level			
Low		*Ref*.	*Ref*.
High		0.39 (0.25‐0.63)	0.49 (0.29‐0.85)**
Community education level			
Low		*Ref*.	*Ref*.
High		2.07 (1.44‐2.99)	1.31 (0.85‐2.01)

Predictors at *p*‐value < 0.05 = *, <0.01 = ** and <0.001 = ***

## 4 DISCUSSION

This study aimed to identify predictors of a minimum acceptable diet among children aged 6‐23 months. The result of the final analysis revealed that 10.44% of the children meet the minimum acceptable diet. Besides, child age, mother's educational level, number of children in the household, place of delivery, having a postnatal checkup 2 months after delivery and community poverty level were predictors of a minimum acceptable diseases.

The finding of this study was lower than the study done in Nepal (29%) (K. C. et al., 2023), Indonesia (47.7%) (Dewanti et al., 2015) and Congo (33%) (Kambale et al., 2021). In contrast, the finding is higher than the study done in India (8.4%) (Acharya et al., 2021), Uganda (5.34%) (Kimuli et al., 2023) and Guinea (3.10%) (Aboagye et al., 2021). However, the finding consistent with the study done in east Africa (11.56%) (Worku et al., 2022). The observed discrepancy in the data could be attributed to a combination of factors, including differences in geographical location, population growth rates, and socioeconomic conditions across the countries being compared (Lutter et al., 2011). Besides, people's cultural background and understanding of an adequate diet can significantly impact their feeding habits (Gizaw & Tesfaye, 2019). Governmental efforts to implement national nutrition programs regarding adequate feeding practices might also have a significant influence (Ahoya et al., 2019). Furthermore, introducing the Baduta program by the Global Alliance for Improved Nutrition (GAIN) has improved achieving minimum acceptable diet in Indonesia (Mallipu, 2021) and such a program may also be of benefit in Ethiopia.

Increasing educational attainment mosre than doubles the likelihood of achieving a minimum acceptable diet (MAD) among mothers who attend primary and secondary education. Several other studies also report a positive association between mother's educational level and meeting MAD (Feleke & Mulaw, 2020; Molla et al., 2021; Senarath et al., 2012). This could be due to the fact that higher education leads to better knowledge of nutrition and the consequences of inadequate feeding, thereby enabling mothers to achieve MAD for their children.

As age increased from 6 to 23 months, the likelihood of achieving a minimum acceptable diet decreased. The conclusions drawn from this study concur with those reported in earlier studies (Sapkota et al., 2022; Tassew et al., 2019). Between the ages of six and twelve months, infants face challenges in consuming solid foods and may develop picky eating habits influenced by various factors. During this critical developmental stage, infants are still refining their oral‐motor skills, which can lead to difficulties in effectively chewing and swallowing solid foods. Additionally, the transition from exclusive breastfeeding or formula feeding to solid foods exposes infants to new textures and flavors, requiring adaptation to unfamiliar sensations. Natural neophobia, coupled with sensory sensitivities, contributes to selective eating habits as infants become more discerning about their food preferences. Parental feeding practices also play a significant role, with pressure to eat potentially exacerbating picky eating behaviors. Moreover, as children age increases, their increased nutritional requirements pose additional challenges, particularly for families in low socioeconomic settings who may struggle to afford adequate nutritious foods, further complicating efforts to achieve a minimum acceptable diet. In addition, they are more susceptible to illnesses that can disrupt their appetite, hindering their ability to achieve a minimum acceptable diet.

This study found a negative association between the number of children in the household and meeting MAD. Likewise, in another study, an increase in the number of children reduces the likelihood of attaining MAD (Pranita et al., 2023). The increasing number of children in a household can pose financial challenges and increase food demand, making it difficult to afford a diverse diet and maintain adequate meal frequency. This can lead to an inability to meet the minimum acceptable diet (MAD) for many children in the household.

Placed of delivery was also found to be an independent predictor of MAD. Mothers who gave birth in health institutions were more than one and a half times more likely to achieve the MAD. This finding is supported by other studies (Birie et al., 2021; Guirindola et al., 2018). This could be because mothers who deliver in health care settings have a better chance to be instructed by health care professionals towards adequate feeding practices for the infant. Besides, delivering a baby in a healthcare facility can lead to more interactions with healthcare professionals, which increases the likelihood that mothers will receive information about the potential consequences of inadequate feeding for their child. In Ethiopia, the prevalence of home delivery is high (66.7%) (Ayenew et al., 2021). Regardless of its immediate impact on mothers and newborns, home delivery has also been linked to inadequate feeding practices later in the child's life (Issaka et al., 2015), making it challenging to accelerate progress toward ending child malnutrition. Therefore, encouraging mothers to give birth in healthcare settings is paramount.

Having a PNC checkup within 2 months after delivery increases the chance of meeting minimum acceptable dietary requirements. Other studies have also arrived at the same conclusion (Abebe et al., 2021; Gizaw & Tesfaye, 2019). PNC checkups provide an opportunity for healthcare providers to personalize their counseling and support to mothers. By addressing specific concerns, dispelling misconceptions, and offering tailored advice on feeding practices and dietary needs, providers empower mothers to make informed decisions that promote a minimum acceptable diet for their babies.

Furthermore, children from high community poverty levels are less likely to get the minimum acceptable diet. The findings of this study align with those of earlier research (Guirindola et al., 2018; Kambale et al., 2021). In communities experiencing high levels of poverty, several interconnected factors contribute to the challenge of ensuring adequate and healthy food for children. These factors include limited financial resources, resources for preparing healthy meals, limited storage facilities, cultural influences, and food preferences (Monterrosa et al., 2020; Wiig Dammann & Smith, 2009). Thus, addressing income inequality and poverty reduction should be the area of concern.

This study uses nationally representative data. Thus, it can be generalizable at a country level and have better statistical power. The secondary nature of the data hinders to explore further predictors of a minimum acceptable diet. Since the data were cross‐sectional, it is difficult to draw a causality relationship between the explanatory and outcome variables.

## 5 CONCLUSION

Based on the findings presented, it is evident that urgent intervention is needed to address the low prevalence of a minimum acceptable diet among children in Ethiopia, which stands at only 10.44%. This figure falls significantly below both regional and global averages (16%) (UNICEF DATA, 2015), emphasizing the severity of the situation and the pressing need for action.

To effectively address this issue, it is essential to target specific factors identified as predictors of a minimum acceptable diet. Child age emerges as a critical consideration, highlighting the importance of focusing on nutritional interventions during the crucial early years of life, particularly for children aged one to 2 years old. Furthermore, maternal education is identified as a significant predictor, suggesting that efforts to improve female education can have a substantial impact on child nutrition outcomes. Community mobilization initiatives aimed at enhancing access to education for girls and women should, therefore, be prioritized as part of broader efforts to improve child nutrition. Additionally, interventions aimed at improving maternal healthcare utilization, such as promoting institutional delivery, family planning utilization, and increasing access to postnatal care services, are recommended. Addressing underlying socioeconomic factors, including poverty and economic instability, is also crucial. Programs and policies aimed at promoting overall economic development and poverty reduction efforts are essential for creating an enabling environment where families can afford and access nutritious food for their children.

## AUTHORS' CONTRIBUTIONS

Tegene Atamenta kitaw originate the idea; formulate the protocol, acquisition of data analysis and interpretation. Befkad Derese Tilahun data analysis and editing the manuscript. Biruk Beletew Abate analysis, methodology, review and editing the manuscript. Ribka Nigatu Haile data curation, formal data analysis and manuscript write up. The author's consented to submit the article to the current journal.

## CONFLICT OF INTEREST STATEMENT

The authors declare no conflicts of interest.

## CONSENT FOR PUBLICATION

None.

## Data Availability

The data that support the findings of this study are available from the corresponding author upon reasonable request.
